# Emotional Exhaustion Variables in Trainee Teachers during the COVID-19 Pandemic

**DOI:** 10.3390/ejihpe13020021

**Published:** 2023-01-24

**Authors:** Jonathan Martínez-Líbano, María-Mercedes Yeomans

**Affiliations:** 1 Facultad de Educación y Ciencias Sociales, Universidad Andrés Bello, Santiago 7591538, Chile; 2 Facultad de Salud y Ciencias Sociales, Universidad de Las Américas, Viña del Mar 2520000, Chile

**Keywords:** emotional exhaustion, mental health, trainee teachers, pandemic

## Abstract

Introduction: emotional exhaustion among trainee teachers is a relevant topic since it could have repercussions regarding the lives of their future pupils. Our objective was to determine the degree of trainee teachers’ emotional exhaustion and associated variables during the COVID-19 pandemic. Methods: the design was cross-sectional and descriptive. Questionnaires with sociodemographic variables, perceptions of mental health, and the Emotional Exhaustion Scale (ECE) (α = 0.890; ω = 0.893) validated for the Chilean context were answered by 204 trainee teachers. The results were analyzed using SPSS software version 25 and the Emotional Exhaustion Interpretation Table (EES-Int). Results: the results show that 92.2% of the trainee teachers presented a worsening in their mental health, stress (66.2%), irritability (38.2%), anxiety (37.7%), and depressive symptoms (32.8%). Online classes (73.04%) and the pandemic (67.6%) were the main influencing factors. Education students who perceived their mental health had worsened became 6.63 times more likely to develop emotional exhaustion [AOR = 6.63; 95% CI: 1.78, 24.69]. In addition, education students with a high perception of academic stress were 7.45 more likely to develop emotional exhaustion [AOR = 7.45; 95% CI: 1.98, 28.09]. Conclusion: we can conclude that trainee teachers have high levels of emotional exhaustion and their perception of their mental health and the academic stress they are being subjected to during the COVID-19 pandemic may lead them to present symptoms of frequent or permanent problems with concentration, attention, recall of information, dissatisfaction with their performance, and frequent learning difficulties. From the affective dimension, they present frequent or permanent anxiety, restlessness, irritability, indifference, low mood, and psychomatization. From the socio-interactional dimension, they present frequent or permanent social withdrawal, interpersonal problems, problems at work or school, and family and relationship problems. Increasing the sample to delve into emotional exhaustion by subject area is necessary. For future studies, research should be conducted on the causes of emotional exhaustion by subject area and the coping strategies of trainee teachers to understand differences and provide input on emotional support in practice.

## 1. Introduction

The World Health Organization classified the spread of COVID-19 as a global pandemic [1]. As a result, the inescapable focus on the transmission and physical repercussions of COVID-19 in the world is likely to minimize public interest in psychosocial consequences in individuals related to the pandemic [2]. In this regard, the pandemic affected all societies and presented an unprecedented challenge in many areas, such as the environment, economy, education, and mental health [3,4,5,6,7,8]. When people isolate themselves, social support structures break down, and people lose exchanges with other significant elements of their environment, such as family, friends, community, or labor organizations, which usually act as sources of emotional and material support [9]. This can affect mental wellbeing and cause feelings of loneliness, anxiety, and depression [10].

Online teaching is not new. However, transitioning from a face-to-face learning process to a remote one because of a pandemic such as COVID-19 is an element that needs to be studied because of the possible repercussions on both teachers and students. The abrupt and troublesome change was remarkably challenging for students and teachers [5,11]. Cases of higher education having to transition from face-to-face to remote learning processes due to an emergency are rare. There are cases of higher education institutions having the same switch due to emergencies, such as in wildfires [12] and hurricanes [13,14], but the consequences of these processes have not been fully determined. It has been documented that online learning has some problems and virtues. There is a higher dropout rate among the former than traditional instruction [15], and there is a general decrease in participation in these courses [16]. Among its virtues, online courses provide learning results equivalent to their face-to-face counterparts [17] and are used to reach students who would otherwise miss out on receiving an education [3].

This new scenario of uncertainty has provoked different reactions in people, including fear, anguish, irritability, stress, anxiety, anger, the memory of traumas, alterations in concentration, and sleeping problems [18]. A considerable increase in mental health problems has been reported [19], with a high prevalence of anxiety and stress disorders [20]. Some studies indicate that college students are a high-risk group for mental health problems [21]. They must go through a critical period when they experience various stressful events. As education progresses to a higher level, students face greater challenges, such as increasingly complex curricula, assignments, and projects [22]. Also, when entering university, they come from different socioeconomic backgrounds and enter the programs directly from high school [23]. This can be a risk factor for mental health, and six primary risk factors include psychological, academic, biological, lifestyle, social, and financial risk factors, which can have varying degrees of impact on students’ mental health [24]. Even though there is an extensive prevalence of psychological distress affecting students in higher education, existing research on student stress remains mainly theoretical [25]. Students often experience high levels of stress and burnout [26], which seriously jeopardizes their experiences in college [27].

Emotional exhaustion (EE) or burnout occurs when one feels they can no longer give more of themselves—it is the reduction of energy or emotional resources [28]. Burnout is considered a syndrome of EE, reduced personal fulfillment, and depersonalization among people who work with people [29]. Among higher-education students, EE is a major problem and has become a public health issue [30]. Distress in students from EE is as complex as mental distress caused by smoking, alcohol consumption, and an unhealthy lifestyle [30]. Contextual burnout can also extend to depression [31], anxiety [32], dropping out, internet dependence, and dissatisfaction with life and educational pathways [33]. Additionally, there might be consequences, such as desertion, sleep disorders, depression, suicide [7,34], and even symptoms that are typically associated with posttraumatic stress disorder and dissociative symptoms [35]. One concern is using drugs and alcohol to reduce stress [36]. Burnout also directly impacts students’ levels of efficiency and productivity [37] and increases their intention to withdraw from university [25]. A student with a high degree of burnout may study the same number of hours as a student with a low degree of burnout but may simply not be as productive due to the various emotional, behavioral, cognitive, and physiological reactions caused by burnout [37].

Current evidence from various countries and educational systems shows that teachers may also be at risk of presenting burnout symptoms, such as EE, anxiety, and depression [38], and the risk is higher if they teach during a period of a pandemic where demands increase and change abruptly, such as from face-to-face teaching to remote teaching [3]. Teachers’ EE is connected to students’ interests, self-concept, and achievement [39]. Without adequate preparation, teachers are ill-equipped to meet the educational needs of diverse learners [40]. Although empirical evidence has focused on professional teachers, there is little evidence on the consequences of EE and its repercussions on their mental health in pre-service teachers. Wellbeing in pre-service teachers is also relevant since it could affect the lives of their future students [39]. Teachers have great responsibility and influence on their students’ cognitive and psychosocial development [41].

The antecedents led us to the hypothesis that trainee teachers presented emotional exhaustion during the pandemic in different degrees and that it might have led to other mental health problems. Given the importance of EE, the repercussions on the mental health of trainee teachers, and the possible consequences it could have on the future students of these trainee teachers, the objective of this study was to determine the degree of trainee teachers’ emotional exhaustion and its repercussions on their mental health.

## 2. Materials and Methods

This is a quantitative study in which the design was cross-sectional and descriptive.

### 2.1. Participants

The inclusion criteria were university students currently pursuing education careers in any higher education institution over 18 years of age who agreed to participate freely in this research. The survey was conducted during November 2020 and was hosted on the Google Forms platform. There were 204 participants.

### 2.2. Instruments

For this study, we used the Emotional Exhaustion Scale (ECE) [42] and the Emotional Exhaustion Interpretation Table (EES-Int) [43].

Emotional Exhaustion Scale (ECE): this instrument aims to measure emotional exhaustion in higher education students using a Likert scale that measures the following statements in five levels (1. Rarely, 2. Few times, 3. Sometimes, 4. Often, 5. Always): tests or evaluations cause me excessive stress; I think I try too hard for the little I get out of it; I feel down in the dumps, kind of sad, for no apparent reason; there are days when I don’t sleep well because of studying; I have headaches and other discomforts that affect my performance; there are days when I feel more exhausted, and I lack the energy to concentrate; I feel emotionally drained by my studies; I feel tired at the end of the day; working and/or studying with evaluations in mind causes me stress, and I lack time, and I feel overwhelmed by my studies. This instrument was recently validated for the Chilean population [44]. The levels of internal consistency are a Cronbach alpha of 0.89 and a satisfactory item homogeneity (inter-item correlation mean = 0.33). The questionnaire applied electronically includes, as a first step, the signing of informed consent for data collection. Additionally, demographic questions were asked, such as age, gender, and academic program. It also included general questions regarding students’ perceptions of their mental health during the pandemic, such as “I believe my mental health has worsened”, “I feel stressed”, “I feel more irritable”, “I feel more sensitive”, “I feel more depressed”, and “I feel more anxious”.

The Exhaustion Interpretation Table (EES-Int) [43] includes a scale that was created in 2021 to interpret the results of the ECE. The score of the ECE is interpreted through the EES-Int Scale in four levels: low (10–19), medium (20–29), high (30–39), and very high (40–50). It describes the indicators of emotional exhaustion under each score level’s cognitive, affective, and social-interactional processes.

Procedure: the questionnaire was sent electronically to the participants. The participants were asked to give their informed consent. It was also made explicit that their responses were anonymous and voluntary and that the results would only be used for academic purposes. Once the data had been collected, we analyzed the results of this descriptive, cross-sectional study.

The study was conducted under the authorization of the Bioethics Committee of Universidad Andrés Bello under resolution 90660/2020, and the study was performed in accordance with the latest version of the Helsinki declaration.

### 2.3. Data Analysis

The sample was collected through Google Forms and was first exported to Excel to code the variables; then, statistical analysis was performed using SPSS version 25. First, a descriptive analysis of the various variables in the study was performed, followed by a binary logistic regression, to establish the relationships between the independent and dependent variables. After this, the variables with a *p*-value of less than 0.2 were analyzed via multiple logistic regression. Statistical significance was established with a *p*-value less than 0.05. The strength of association was interpreted using the adjusted odds ratio (OR) and 95% CI.

## 3. Results

The results of the study are presented as follows: characterization of the participants, reliability of the instrument, participants’ perceptions of their mental health, factors that contributed to a decline in their mental health, analysis of EE by study area, interpretation of the ECE scores, and emotional exhaustion bivariable and multivariable logistic regression analysis for factors associated with emotional exhaustion in trainee teachers.

### 3.1. Participants

The population consisted of 204 Chilean trainee teachers in higher education, including 161 women (78.9%), 42 men (20.6%), and one non-binary person (0.5%). Among the age ranges, 19.6% were between 18 and 20, 54.4% were between 20 and 29, 20.6% were between 30 and 39, 3.9% were between 40 and 49, and 1.5% were between 50 and 59. Among the trainee teachers and their majors, we found students in Early Childhood Education (19.1%); Bachelor’s Degree in Physical Activity (8.3%); Pedagogy in Biology and Chemistry (0.5%); Pedagogy in Elementary Education (22.5%); Pedagogy in Special Education (21.6%); Pedagogy in Physical Education (9.8%); Pedagogy in History (1%); Pedagogy in English (5.4%); Technical Assistant to Child Educator (2.5%); Technician in Physical Activity and Sports (5.4%); Pedagogy in Biology (2.5%); and Pedagogy in Arts (1.5%).

### 3.2. Instrument Reliability

The reliability of the instrument was measured using Cronbach’s alpha (α = 0.890) and the omega coefficient (ω = 0.893). Based on these results, the Emotional Exhaustion Scale in this sample presented a good reliability index.

### 3.3. Trainee Teachers’ Perceptions of Their Mental Health

Table 1 shows trainee teachers’ perceptions of their mental health during the pandemic. Among the trainee teachers, 92.2% stated that their mental health has worsened, 66.2% stated that they presented symptoms of stress, 38.2% presented irritability, 50% presented greater sensitivity, 37.7% presented anguish and/or anxiety, and 32.8% presented depressive symptoms.

### 3.4. Factors That Contributed to the Decline in Mental Health

The factors that most affected the trainee teachers’ mental health were online classes (73.04%), the pandemic (67.6%), social distancing (49.51%), confinement (43.63%), economic problems (43.1%), and news and media about COVID-19 (23.5%) (Table 2).

### 3.5. Emotional Exhaustion by Study Area

Table 3 shows the major of the future teachers and their level of EE. There were twelve majors represented by the participants. They are positioned in rows, and the number of participants (*n*) and the percentage of participants (%) per major is shown, which are classified by level of EE.

Table 4 shows EE by study area. Participants with majors in Pedagogy in Physical Education and Physical Activity were grouped in the first area. Most trainee teachers presented high or very high EE in all programs. Pedagogies in History, English, and Arts were grouped in arts and humanities. Pedagogies in Biology and Chemistry were grouped in science. Early Childhood Education and Technical Assistant to the Child Educator were grouped in preschooler Education. Pedagogy in Special Education was set in the special education area, and Pedagogy in Elementary Education was set in the elementary education area.

### 3.6. Emotional Exhaustion Scale Interpretation

According to the EES-Int, from the cognitive dimension, 87.7% of trainee teachers manifested symptoms of frequent or permanent problems with concentrating, paying attention, and remembering information, frequent dissatisfaction with their own performance, and frequent learning difficulties. From the affective dimension, the same group presented frequent or permanent anxiety, anguish, irritability, indifference, low mood, and psychomatization. From the socio-interactional dimension, the trainee teachers presented frequent or permanent social withdrawal, interpersonal problems, problems at work or school, and family and relationship problems.

### 3.7. Emotional Exhaustion Bivariable and Multivariable Logistic Regression Analysis for Factors Associated with the Emotional Exhaustion of Trainee Teachers

After performing the descriptive analysis, we proceeded to perform the bivariate analysis. In the bivariate analysis, gender, perception of worsening mental health, perception of academic stress, and hours of study in front of the computer were significantly associated with emotional exhaustion (see Table 5).

After controlling confounders through the multivariable analysis, perception of worsening mental health and perceived academic stress were significantly associated with emotional exhaustion among trainee teachers.

Education students who perceived their mental health had worsened were 6.63 times more likely to develop emotional exhaustion [AOR] = 6.63; 95% CI: 1.78, 24.69]. In addition, education students with a high perception of academic stress were 7.45 more likely to develop emotional exhaustion [AOR] = 7.45; 95% CI: 1.98, 28.09]. Thus, mental health and academic stress could generate greater emotional exhaustion during the COVID-19 pandemic in trainee teachers.

## 4. Discussion

Concerning the reliability of the instrument, the Cronbach’s alpha calculated by the original authors was 0.83 [42]. It was 0.89 when validated for the Chilean context [44]. In other Latin American countries, its reliability was also good; for example, it was 0.85 in Peru [45] and 0.83 in Mexico [46]. The Cronbach’s alpha was 0.89 for the present application on trainee teachers.

This research shows the complexity of the COVID-19 pandemic’s impact on trainee-teachers mental health. It generates high EE, which can cause serious long-term consequences for mental health [47]. The results presented are consistent with a recent systematic review which found that high levels of internet and social network use in university students were correlated with the presence of the fear of missing out (FOMO), anxiety, depression, stress, internet addiction, learning disabilities, and sleep disorders [48].

Education students who perceive their mental health negatively were 6.63 times more likely to develop emotional exhaustion [AOR] = 6.63; 95% CI: 1.78, 24.69]. The above is understandable given that teachers have seen their mental health depleted during the COVID-19 pandemic [49,50] since the teaching profession is stressful and very demanding [51], which has directly impacted the occurrence of burnout in teachers [52].

Students who presented a high perception of academic stress were 7.45 more likely to develop emotional exhaustion [AOR] = 7.45; 95% CI: 1.98, 28.09], which is understandable given that stress can lead teachers to perceive themselves as less capable [53], which impacts their self-efficacy [54] and causes greater burnout [55]. Therefore, we can conclude that mental health and perceived academic stress could generate greater emotional exhaustion during the COVID-19 pandemic in trainee teachers.

Regarding EE levels by area of study, we found that most students in all areas presented high or very high EE (Physical Education and Physical Activity 76.6%; Arts and Humanities 81.25%; Science 100%; Preschool Education 93.18%; Special Education 90.9%; Elementary Education 91.31%). However, the high and very high EE proportions showed some differences. The study areas of Arts and Humanities, Preschool Education, Special Education, and Primary Education presented a higher proportion of very high EE compared to high EE (68.75–12.50%, 61.36–31.82%, 70.45–20.45%, and 65.22–26.09%, respectively). In contrast, in some areas of study, the proportion between very high and high EE was lower at the highest level (36.17–40.43% in the study area of Physical Education and Physical Activity and 28.57–71.43% in the Sciences). This could be explained from two perspectives.

First, several studies have shown that physical activity contributes to mental health in several ways [56,57]. Usually, trainee teachers in this study area are physically active regularly. That is why only 36.4% reached the highest level of EE. This is a tremendous contrast with Special Education trainee teachers, of which 70.5% reached the highest level.

Only 28.6% of trainee teachers reached the highest level regarding the Science Study Area. However, the whole sample of this study area reached a high or very high level of EE. Since a hundred percent of the sample was in a high or very high level of EE, we cannot discuss proper emotional management as we could in the Physical Activity and Physical Education area.

From the cognitive dimension of the EES-Int, 87.7% of the trainee teachers reported symptoms of frequent or permanent trouble concentrating, paying attention, and remembering information, dissatisfaction with their own performance, and learning difficulties. This result coincides with the respondents’ perception regarding their mental health as 92.2% of the sample reported a deterioration of their mental health. The evaluation of the effects of EE on the cognitive area coincides with previous studies where high EE was correlated with various areas in people’s lives, such as job satisfaction, job performance, learning difficulties [58], work stress [59], and feelings of academic incompetence [27]. According to the present study, online classes was the element that most affected trainee teachers regarding the decline in their mental health (73%). Chilean students abruptly switched from face-to-face education to remote education [3,4]. In addition, cognitive and behavioral stress due to the uncertainty of the pandemic [60] has generated a constant shift in specific internal and/or external demands which exacerbate or exceed peoples’ resources [61]. Trainee teachers have had to face the demands of the environment, such as the transition from face-to-face to remote classes, which, according to studies in this context, could have harmful consequences for students [62,63]. Other authors added that feeling emotionally exhausted leads to low productivity and low efficiency [64].

From the affective dimension, the same group presented frequent or permanent anxiety, distress, irritability, indifference, low mood, and psychomatization. In this sample, in addition to perceiving a deterioration of their mental health (92.2%), trainee teachers’ also presented sensitivity (50%), anguish and/or anxiety (37%), and depressive symptoms (32.8%). The COVID-19 pandemic has significantly affected the general population’s quality of life and mental health. Some research reports that during the outbreak, people with no psychiatric history who were diagnosed with generalized anxiety disorder increased by 12%, and people with no psychiatric history who were diagnosed with depression increased by 29% [65]. These results are consistent with other research where university students’ perception of their mental health was diminished by the COVID-19 pandemic, especially in those with pre-existing pathologies [66,67]. Some studies show that negative affectivity plays a role in EE development (or the tendency to feel depression, anxiety, or stress) [6]. However, while teachers with a more anxious profile show a greater EE, those with a depressive profile find it more challenging to develop a strong sense of personal achievement [68]. EE can lead to several adverse outcomes, such as depression, social isolation, and physical health consequences [69]. Consequently, in our study, 32.8% of the participants reported having depressive symptoms. Likewise, stress is associated with poor mental health, resulting in significant EE and psychological distress [70]. This is consistent with our results, where 66.2% of the participants reported experiencing stress, and 37.7% of them reported experiencing anxiety or distress during the COVID-19 pandemic, which could also be related to the high levels of emotional fatigue in this sample.

From the socio-interactional dimension, trainee teachers presented frequent or permanent social withdrawal, interpersonal problems, problems at work or school, and family and couple problems. Conflict within the family, worsening of mental health [71,72] and high levels of EE drastically reduce peoples’ sense of wellbeing and increase their risks of developing depression, substance abuse disorder, heart disease, and somatic symptoms [73]. There is a negative relationship between EE and empathy. Therefore, those with high EE tend to be less empathetic [74]. Lack of empathy could generate problems for future teachers when relating to their environment and the prospective students of these trainee teachers. This point is relevant since empathy is fundamental to achieving student learning results [75]. Empathy is often cited as an essential characteristic of teachers which enables adequate communication between the participants in the educational process. Therefore, emotional competencies are crucial to successfully carry out the various professional roles of teachers [76]. In our study, 43.6% of the participants reported that confinement had influenced the decline in their mental health, and 49.5% reported that social distancing had also affected this decline.

The present research results should draw the attention of political and academic authorities to develop effective intervention strategies for these trainee teachers, given that their mental health could have repercussions for their future students in the schools where they grow professionally.

## 5. Conclusions

As for the main objective, which was to determine the degree of emotional exhaustion and its repercussions on the mental health of trainee teachers, we have concluded that 87.7% presented high or very high levels of pandemic emotional exhaustion.

These prospective teachers’ perceptions of their mental health and academic stress were significantly associated with emotional exhaustion during the COVID-19 pandemic.

From the cognitive dimension, the repercussions were that 87.7% of trainee teachers showed symptoms of frequent or permanent problems in concentrating, paying attention, and remembering information, dissatisfaction with their performance, and frequent learning difficulties. From the affective dimension, they presented frequent or permanent anxiety, anguish, irritability, indifference, low mood, and psychomatization. Furthermore, from the socio-interactional dimension, they presented frequent or permanent social withdrawal, interpersonal problems, problems at work or school, and family and relationship problems.

Practical applications of these results include drawing the attention of government and academic authorities and those who train these teachers. The effects of the pandemic could influence the emotional distress of these future teachers and perpetuate harmful impacts on their mental health and on their prospective students in the various educational establishments where they will work.

This study has some limitations. It is necessary to increase the sample to delve into emotional exhaustion by subject area. For future studies, research should be conducted on the causes of emotional exhaustion by subject area and the coping strategies of trainee teachers to understand differences and provide input on emotional support in practice.

## Figures and Tables

**Table 1:** Trainee teachers’ perceptions of their mental health.

Trainee Teachers’ Perceptions of Their Mental Health	*n*	%
Worsening of Mental Health		
Yes	188	92.2
No	16	7.8
Stress Symptoms		
Yes	135	66.2
No	69	33.8
Irritability Symptoms		
Yes	78	38.2
No	126	61.8
Increased Sensitivity		
Yes	102	50.0
No	102	50.0
Symptoms of Distress and/or Anxiety		
Yes	77	37.7
No	127	62.3
Depressive Symptoms		
Yes	67	32.8
No	137	67.2

**Table 2:** Factors that contributed to trainee teachers’ decline in mental health.

Factors that Contributed to Trainee Teachers’ Decline in Mental Health	*n*	%
Pandemic		
Yes	138	67.6
No	66	32.4
Confinement		
Yes	89	43.63
No	115	56.37
Economic Problems		
Yes	88	43.1
No	116	56.9
Social Distancing		
Yes	101	49.51
No	103	50.49
News and Media		
Yes	48	23.5
No	156	76.5
Online Classes		
Yes	149	73.04
No	55	26.96

**Table 3:** Emotional exhaustion by major.

Major		Low EE	Medium EE	High EE	Very High EE
Early Childhood Education	*n*	0	2	12	25
	%	0	5.1	30.8	64.1
Bachelor’s Degree in Physical Activity	*n*	1	3	9	4
	%	5.9	17.6	52.9	23.5
Pedagogy in Biology and Chemistry	*n*	0	0	1	0
	%	0	0	100	0
Pedagogy in Elementary Education	*n*	1	3	12	30
	%	2.3	6.8	27.3	68.2
Pedagogy in Special Education	*n*	1	3	9	31
	%	2.3	6.8	20.5	70.5
Pedagogy in Physical Education	*n*	1	5	3	11
	%	5	25	15	55
Pedagogy in History	*n*	0	1	0	1
	%	0	50	0	50
Pedagogy in English	*n*	1	1	1	8
	%	9.1	9.1	9.1	72.7
Technical Assistant to the Child Educator	*n*	0	1	2	2
	%	0	20	40	40
Physical Activity and Sports Technician	*n*	1	0	8	2
	%	9.1	0	72.7	18.2
Pedagogy in Biology	*n*	0	0	3	2
	%	0	0	60	40
Pedagogy in Arts	*n*	0	0	1	2
	%	0	0	33.3	66.7
Total	*n*	6	19	61	118
	%	2.9	9.3	29.9	57.8

**Table 4:** Emotional exhaustion by study area.

Study Area	N	Low EE	Medium EE	High EE	Very High EE
Physical Education and Physical Activity	47	3	8	19	17
		6.38%	17.02%	40.43%	36.17%
Arts and Humanities	16	1	2	2	11
		6.25%	12.50%	12.50%	68.75%
Science	7	0	0	5	2
		0%	0%	71.43%	28.57%
Preschooler Education	44	0	3	14	27
		0%	6.82%	31.82%	61.36%
Special Education	44	1	3	9	31
		2.28%	6.82%	20.45%	70.45%
Elementary Education	46	1	3	12	30
		2.17%	6.52%	26.09%	65.22%

**Table 5:** Emotional exhaustion bivariable and multivariable logistic regression analysis for factors associated with emotional exhaustion in trainee teachers.

Variables	Category	Emotional Exhaustion		COR (95%CI)	AOR (95%CI)
		Yes (%)	No (%)		
Sex	Male	43 (84%)	7 (16%)	1	1
	Female	155 (96%)	6 (4%)	0.19 (0.06, 0.62)	5.02 (1.59, 15.85)
Fear of getting infected	Yes	133 (95%)	7 (5%)	1	1
	No	58 (91%)	6 (9%)	0.50 (0.16, 1.58)	0.50 (0.16, 1.58)
Perception of mental health worsening	Yes	179 (95%)	9 (5%)	1	1
	No	12 (75%)	4 (25%)	0.15 (0.04, 0.56)	6.63 (1.78, 24.69) *
Academic stress perception	Yes	132 (98%)	3 (2%)	1	1
	No	59 (82%)	13 (18%)	0.13 (0.036, 0.50)	7.45 (1.98, 28.09) *
PC Study Hours	>5 h	70 (91%)	7 (9%)	1	1
	<5 h	121 (95%)	6 (5%)	2.01 (0.652, 6.23)	0.49 (0.160, 1.53)

* *p* < 0.005 and 1: constant.

## Data Availability

Data sets that were analyzed or generated during the study are available for sharing as needed.
